# A diagnostic marker kit for Fusarium wilt and sterility mosaic diseases resistance in pigeonpea

**DOI:** 10.1007/s00122-020-03702-0

**Published:** 2020-10-20

**Authors:** Rachit K. Saxena, Anil Hake, Abhishek Bohra, Aamir W. Khan, Anupama Hingane, Rafat Sultana, Indra Prakash Singh, S. J. Satheesh Naik, Rajeev K. Varshney

**Affiliations:** 1 International Crops Research Institute for the Semi-Arid Tropics (ICRISAT), Patancheru, Hyderabad, Telangana India; 2 ICAR- Indian Institute of Pulses Research (IIPR), Kanpur, Uttar Pradesh India; 3 Bihar Agricultural University, Sabour, Bhagalpur, Bihar India

## Abstract

Fusarium wilt (FW) and sterility mosaic diseases (SMD) are key biotic constraints to pigeonpea production. Occurrence of these two diseases in congenial conditions is reported to cause complete yield loss in susceptible pigeonpea cultivars. Various studies to elucidate genomic architecture of the two traits have revealed significant marker–trait associations for use in breeding programs. However, these DNA markers could not be used effectively in genomics-assisted breeding for developing FW and SMD resistant varieties primarily due to pathogen variability, location or background specificity, lesser phenotypic variance explained by the reported QTL and cost-inefficiency of the genotyping assays. Therefore, in the present study, a novel approach has been used to develop a diagnostic kit for identification of suitable FW and SMD resistant lines. This kit was developed with 10 markers each for FW and SMD resistance. Investigation of the diversity of these loci has shown the role of different alleles in different resistant genotypes. Two genes (*C*.*cajan_03691* and *C.cajan_18888*) for FW resistance and four genes (*C*.*cajan_07858, C*.*cajan_20995, C*.*cajan_21801* and *C*.*cajan_17341*) for SMD resistance have been identified. More importantly, we developed a customized and cost-effective Kompetitive allele-specific PCR genotyping assay for the identified genes in order to encourage their downstream applications in pigeonpea breeding programs. The diagnostic marker kit developed here will offer great strength to pigeonpea varietal development program, since the resistance against these two diseases is essentially required for nominating an improved line in varietal release pipeline.

**Electronic supplementary material:**

The online version of this article (10.1007/s00122-020-03702-0) contains supplementary material, which is available to authorized users.

## Introduction

Since the inception of breeding, it has been defined as a combination of “Science” and “Art”. The “Science” component mainly includes understanding of genetics for desirable traits in a particular crop, whereas “Art” has relied on a breeder’s experience. Therefore, knowledge of genetics and the experience of breeder both remain essential to breed an improved cultivar/variety. A number of improved crop varieties resulting from plant breeding have contributed significantly to global food production. In spite of the significant progress in conventional breeding to date, it has been realized as a herculean task to match with the dietary demands of continuously growing population in changing climate. The major reason is limited advancement in genetic gains in most of the crops species by following conventional approaches. Therefore, it is high time to focus on different components of the genetic gains/breeder’s equation [genetic gain (∆*G*) = heritability (*h*^2^) × phenotypic variability in population (*σ*_p_) × selection intensity (i)/generation interval (*L*)] in almost all the crop improvement programs and transform conventional breeding to data-driven efficient and predictive breeding.

Over the last few decades, remarkable advances in genomics have provided improved tools for enhancing the efficiency in genetic improvement. Particularly, molecular markers have been applied in crop improvement programs through genomics-assisted breeding (GAB). As a result, a number of crop varieties developed through GAB were released in rice (Yang et al. 2019; Yugander et al. 2018), maize (Krishna et al. 2017; Muthusamy et al. 2014), barley (Sayed and Baum 2018; Mikołajczak et al. 2016), wheat (Randhawa et al. 2019; Tyagi et al. 2014), chickpea (Mannur et al. 2019; Varshney et al. 2013, 2014a), groundnut (Kolekar et al. 2017; Varshney et al. 2014b), etc., whereas in many other crop species, the potential of GAB is yet to be realized. It is also true in the case of pigeonpea (*Cajanus*.* cajan*), one of the most important pulse crops and chief sources of protein to the vegetarian population especially in India. The slow progress of pigeonpea improvement may be attributed to a number of factors, which include its inherent nature such as generation time, sensitivity to diseases and photoperiod, low level of genetic diversity, low priority of the policy makers, etc. Equally important factors also include lack of diagnostic DNA markers for breeding applications. In pigeonpea during last one decade, a number of genomic resources including draft genome (Varshney et al. 2012), whole-genome sequencing (WGS) data (Kumar et al. 2016; Varshney et al. 2017), several genotyping platforms such as 56 K *Cajanus* SNP genotyping array (Saxena et al. 2018), genetic maps (Saxena et al. 2012; Bohra et al. 2012) and quantitative trait loci (QTLs)/markers associated with traits (Saxena et al. 2017a, b, c, 2018, 2020; Yadav et al. 2019; Obala et al. 2019; Singh et al. 2016) have been identified. In the case of the two most important diseases in pigeonpea, i.e. Fusarium wilt (FW) and sterility mosaic diseases (SMD), available markers identified so far are not very effective for selection in breeding. On the other hand, resistance to both diseases is an essential prerequisite for nomination of any advanced breeding line for release of a new variety in major pigeonpea growing countries like India.

Key challenges that the GAB for resistance breeding in pigeonpea faces include genetically complex nature of these diseases, a variety of strains/isolates/variants/pathotypes, limited availability of disease resistant sources, inadequate phenotypic variability in segregating populations, low-resolution genetic maps and QTLs with low phenotypic variations explained (PVE). As mentioned above, initial efforts in molecular mapping of FW and SMD were not much successful (Kotresh et al. 2006; Gnanesh et al. 2011). Subsequently, with the advantage of draft genome sequence information (Varshney et al. 2012), novel approaches based on early generations such as F_2_s and WGS of bulked samples were used to develop markers (SNPs and Indels) for FW and SMD (Singh et al. 2016, 2017). More recently, comprehensive approach using multiple segregating populations (RILs and F_2_s) and genotyping by sequencing (GBS) was used for molecular mapping of FW and SMD (Saxena et al. 2017a, b, c). Though these studies have provided a number of genomic regions associated with FW and SMD resistance, diagnostic markers could not be developed for breeding applications. In the present study, we have applied a novel approach for the development of diagnostic markers for FW and SMD resistance in pigeonpea. In brief, we have knitted a number of datasets from different studies and consolidated positive signals or associated genomic regions to useful diagnostic marker kit for the selection of FW and SMD resistance in pigeonpea.

## Methods

### Plant materials

Two sets of pigeonpea genotypes including first set of 21 genotypes (7 susceptible and 14 resistant) for FW response and a second set of 13 genotypes (3 susceptible and 10 resistant) for SMD response were selected based on the availability of phenotyping and genotyping data (Supplementary Tables 1, 2, 3). Above-mentioned genotype sets were used for discovery of the most potential DNA markers and their initial phenotypic validation. Further, 74 pigeonpea genotypes including parents of segregating populations, released varieties and elite breeding material and BC_1_F_1_ individuals were used for the validation of genotyping assays (Supplementary Table 4). Selected BC_1_F_1_s were developed from parental combination of recipient parents BDN 711 crossed with donor ICPL 20096 to generate F_1_s. The F_1_s resulting from crossing were used as the pollen parent and crossed with recipient parent, i.e. BDN 711 to generate BC_1_F_1_s.

### Sequence variations and data mining

**Fig. 1:**
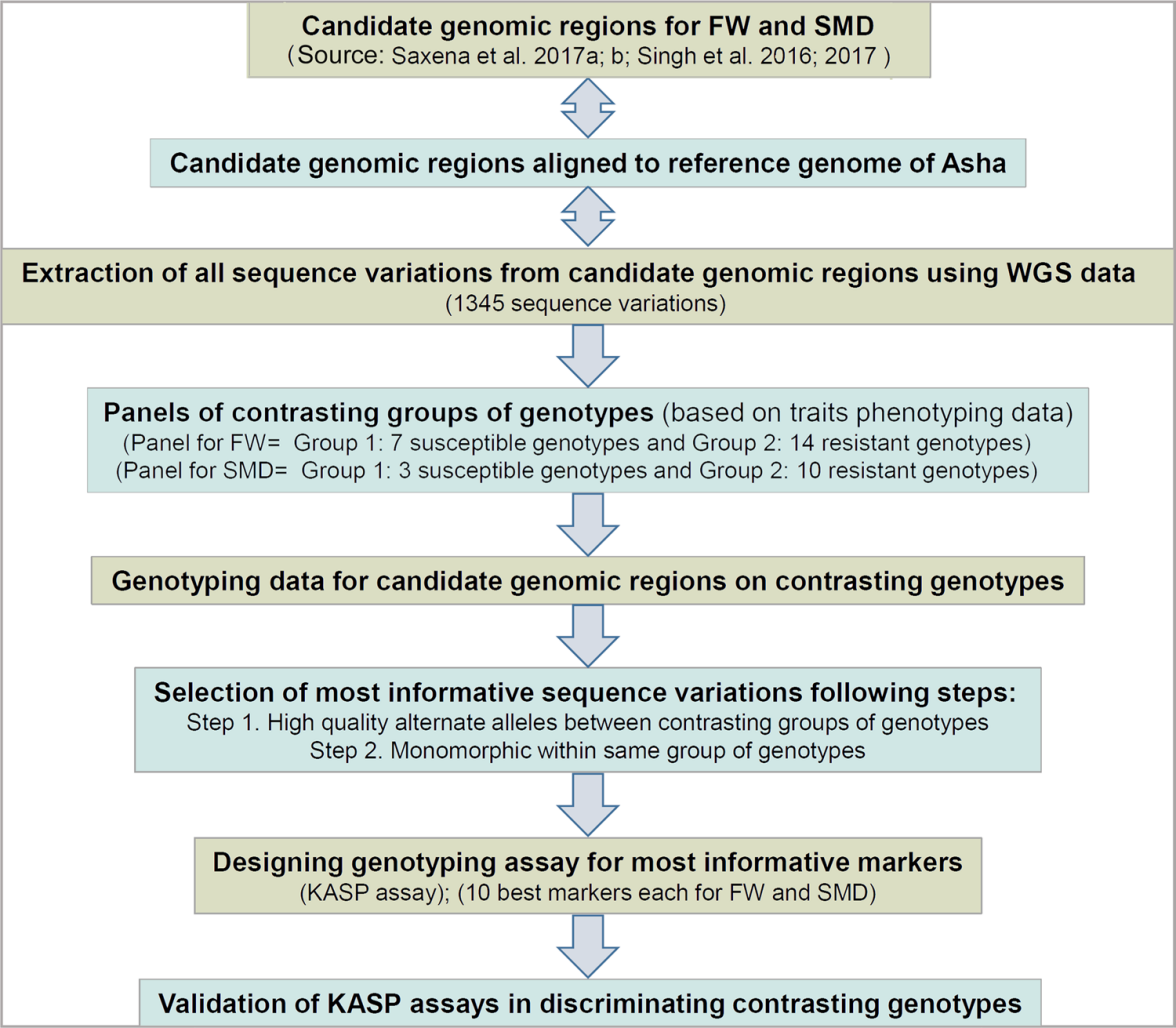
A scheme followed in the present study to identify informative/ diagnostic markers associated with resistance to Fusarium wilt (FW) and sterility mosaic diseases (SMD). “WGS”: whole-genome sequencing; “KASP”: Kompetitive allele-specific PCR

Information on candidate genomic regions was collected from the previous studies focused on the identification of quantitative trait loci (QTL) regions and associated markers with FW and SMD (Singh et al. 2016, 2017; Saxena et al. 2017a, b, c) (Fig. 1). Trait-associated genomic regions were used to pinpoint the sequence variations from available WGS data on 104 pigeonpea lines (unpublished) using the Unified Genotyper of GATK version 4.0 (DePristo et al. 2011). In order to define sequence variation, Phred quality score for the base was ≥ 30 and the number of sequence reads aligned in each of the lines against the reference genome was ≥ 5. Further, only one sequence variant was reported if two or more sequence variants were present in a 5-bp window. These filtered sequence variations were combined with other sequence variations and used in the development of Axiom *Cajanus* SNP Array (Saxena et al. 2018). Further, available array genotyping data and WGS data from the above-mentioned trait-specific sets for FW and SMD were used for data mining using criteria: first, sequence variation should clearly distinguish (high-quality alternate alleles) between the two contrasting (susceptible and resistant) groups of a given trait-specific set, i.e. FW/SMD, and secondly, the selected sequence variation should exhibit the same allelic pattern (monomorphic) among entire genotypes of the same group (Fig. 1).

### DNA extraction

The leaf samples were collected from individual plants of pigeonpea genotypes and BC_1_F_1_s. The leaf samples were supplied to Intertek AgriTech (https://www.intertek.com/agriculture/agritech/) for genomic DNA isolation, quantification and genotyping as a part of the High Throughput Genotyping (HTPG) project (https://cegsb.icrisat.org/high-throughput-genotyping-project-htpg/).

DNA was isolated following the protocol used at Intertek AgriTech based on LGC oKtopure™ automated high-throughput ‘sbeadex™’ DNA extraction and purification system (https://www.biosearchtech.com/). In brief, this protocol homogenises leaf samples by steel bead grinding in 96-deep-well plates. Further homogenised samples were treated with an extraction buffer in the ‘sbeadex™’ kit (https://www.biosearchtech.com/) from LGC. Extracted DNA was purified using superparamagnetic particles coated with ‘sbeadex™’ surface chemistry that captures nucleic acids from a sample. Purified DNA was eluted and used for quantification, dilutions and genotyping.

### Kompetitive allele-specific PCR (KASP) genotyping assay

To develop KASP genotyping assays for target SNPs, flanking sequences of 35–50 bases on either sides of the target SNPs were selected. Flanking sequences were filtered so that it should not have any other SNP, Indel, N or other alphabet (apart from *A*, *T*, *C*, *G*) with a minimum threshold of Q30 and GC proportion between 0.3 and 0.7. Targeted SNPs were selected in such a manner so that it should not present in repeated regions. Following the above-mentioned parameters, KASP assays for target SNPs were designed (Supplementary Table 5). Subsequently SNP genotyping was conducted using KASP assays (https://www.lgcgroup.com/).

### Diversity analysis

SNPs were used to assess polymorphism information content or PIC values, gene diversity across the germplasm by using PowerMarker software (Liu and Muse 2005; https://statgen.ncsu.edu/powermarker/). To cluster the genetic variation, we also performed a principal component analysis in DARwin 6.0.14 (Perrier and Jacquemoud-Collet 2006). Pairwise relatedness was calculated as genetic distance with GenAlEx 6.5 (Peakall and Smouse 2012). The matrix of genetic distances was used to create a neighbour-joining tree with DARwin 6.0.14 (Perrier and Jacquemoud-Collet 2006).

## Results

### Candidate genomic regions and sequence variations

The genomic information on candidate regions associated with FW resistance and SMD resistance was assembled from the four different studies. Namely, QTL regions from Saxena et al. 2017a, b, candidate SNPs from QTL-seq and non-synonymous SNPs from Singh et al. 2016 and insertion and deletion (Indel) markers from Singh et al. 2017 were collected (Fig. 1). The QTL-containing genomic regions as delineated by flanking SNPs in these studies were used for further analysis in order to capture corresponding complete sequence in the draft genome (Varshney et al. 2012). The complete sequence information of the QTL regions, candidate SNPs and Indels thus obtained were used to find out the sequence variations from the available WGS data on 104 cultivated pigeonpea lines generated in another project (unpublished). As a result, 1345 sequence variations were identified across the WGS data from 104 lines within the candidate genomic regions associated with FW and SMD (Supplementary Table 6). These sequence variations include 80.6% (1084) SNPs and 19.4% (261) Indels (Table 1). In line with the distribution of candidate genomic regions, the sequence variations spanned the entire genome. However, the maximum sequence variations (20.07%) were present on CcLG11 and minimum (0.82%) on CcLG05 with an average of 122.3 per CcLG. Of 1345 variations, 47.43% (638) were categorized into intergenic regions, 14.42% (194) as intronic and 38.14% (513) as exonic regions. Within the exonic regions, there were 115 synonymous SNPs (sSNPs) and 383 non-synonymous (nsSNPs) mutations. Other sequence variant types identified included missense variant and splice region variant (6), splice region variant and intron variant (8) and splice region variant and stop retained variant (1). Overall, a total of 502 genes containing the sequence variants were detected across the candidate genomic regions.

**Table 1:** Details on sequence variations identified in the candidate genomic regions for Fusarium wilt and sterility mosaic diseases resistance

CcLGs	Sequence variations in candidate genomic regions		
	Total	Indels	SNPs
CcLG01	58	12	46
CcLG02	253	17	236
CcLG03	86	25	61
CcLG04	68	7	61
CcLG05	11	7	4
CcLG06	167	39	128
CcLG07	139	14	125
CcLG08	150	46	104
CcLG09	22	0	22
CcLG10	121	37	84
CcLG11	270	57	213
Total	1345	261	1084

### Mining of the most informative SNPs/Indels

In order to pinpoint the most informative sequence variations from the total 1345 sequence variations identified in the candidate genomic regions, two sets of genotypes with known reactions to FW (Supplementary Table 1, 2) and SMD (Supplementary Table 1, 3) were developed. The individuals within a given set were categorized into susceptible and resistant groups based on the historical trait phenotyping data. The phenotyping data were collected for 1–18 years per genotype at 1 to 19 locations (Supplementary Table 1, 2, 3). Phenotyping data supported the stable performance of the genotypes in terms of disease reactions in specific location across years (Supplementary Table 2, 3). The phenotyping data also provided information on complex nature of these diseases probably due to different pathogen variants at different locations. Though we did not have the clear idea on the pathogen variability in these diseases, the phenotyping data suggested the presence of location-specific resistant/susceptible genotype. Hence, both types of resistant and susceptible genotypes showing constant diseases reaction at specific location or across the locations were considered. The genotypic information on above-mentioned sets of genotypes was assembled from *Cajanus* 56 K SNP array data (Saxena et al. 2018) or WGS-based studies (Kumar et al. 2016; Varshney et al. 2017; unpublished data on 104 lines). In brief, the historical phenotyping data were used to define contrasting genotype groups, while genotyping data were used to find out the most informative sequence variations within the candidate genomic regions.

#### Diagnostic SNPs/Indels for Fusarium wilt (FW)

In the case of FW, 21 genotypes including 7 susceptible and 14 resistant genotypes were constituted a primary panel to search for the most suitable markers (Supplementary Table 1, 2). It is important to mention that FW causing pathogen, i.e. *Fusarium oxysporum* f. sp. *udum*, has a number of isolate forms in different pigeonpea growing zones (Purohit et al. 2017; Padinhare et al. 2017). However, these isolates have not been characterized at the genome level, but their presence in different growing zones is well known and documented (Naik et al. 2017). Considering this, primary panels of genotypes were selected in such a way that these can represent maximum pigeonpea growing zones with known resistant and susceptible genotypes. As mentioned earlier, a total of 1345 sequence variations from the candidate regions were examined in groupwise manner using criteria: first, sequence variation (high-quality alternate alleles) should clearly distinguish the susceptible and resistant groups of a given set; and secondly, the selected sequence variation should show same allele in all the genotypes from the same group. Out of 1345 sequence variations, 233 were monomorphic across all 21 genotypes, thus making them non-informative (Table 2). We targeted 305 sequence variations that had monomorphic pattern across all susceptible genotypes (7) but had a contrasting allele at least in one resistant genotype, whereas remaining 795 sequence variations were polymorphic across susceptible group of 7 genotypes. In the case of resistant group of 14 genotypes, only 21 monomorphic and 1091 polymorphic sequence variations were identified (Table 2). Taken together, we considered 305 sequence variations showing similar allele in susceptible genotypes and alternate allele in at least one resistant genotype for further analysis. However, we could not identify any sequence variation showing similar allele in all 14 resistant genotypes for any of 305 sequence variations. Nevertheless, it is not unexpected given the variable nature of FW in pigeonpea genotypes. Not one but different alleles might be contributing to the resistance in different genotypes. Therefore, a grading score was developed to further narrow down 305 sequence variations based on the maximum resistant genotypes carrying alternate allele. Following grading score, we could select a set of 10 sequence variations, of which top 5 sequence variations had alternate allele in 11 out of the total 14 resistant genotypes, while remaining 5 sequence variations showed alternate allele in 9 (2), 8 (2) and 7 (1) resistant genotypes (Supplementary Table 7). Of these 10 top ranked sequence variations, 6 were present in the candidate genomic regions of CcLG11 identified earlier (Saxena et al. 2017b; Singh et al. 2016, 2017), whereas one each from the remaining selected sequence variations was present in genomic region on CcLG04, CcLG07, CcLG08 and CcLG10. The functional annotation of selected 10 sequence variations assigned 5 each to intergenic and genic variations. Within the genic variations, 4 (3 synonymous and 1 missense) were present in one gene “*C*.*cajan_03691*” on CcLG11 and remaining one variation found in gene “*C.cajan_18888*” on CcLG07 (Supplementary Table 7).

**Table 2:** Distribution of sequence variations in different sets of genotypes for Fusarium wilt and sterility mosaic diseases

	FW		SMD	
	No. of genotypes	No. of markers	No. of genotypes	No. of markers
Monomorphic across genotypes	21	233	13	139
Monomorphic across susceptible genotypes	7	305	3	699
Polymorphic across susceptible genotypes		795		507
Monomorphic across resistant genotypes	14	21	10	309
Polymorphic across resistant genotypes		1091		897

#### Diagnostic SNPs/Indels for sterility mosaic diseases (SMD)

For SMD, a set of 13 genotypes including 3 susceptible and 10 resistant genotypes was considered as a primary panel (Supplementary Table 1, 3). Like FW, SMD is also a complex disease since the causal agent, i.e. pigeonpea sterility mosaic virus (PPSMV), has several variants in different pigeonpea growing zones (Kumar et al. 2017; Reddy et al. 1993). Therefore, primary panel of genotypes was selected to represent maximum pigeonpea growing zones with known resistant and susceptible genotypes. A total of 1345 sequence variations from the candidate regions were analyzed in groupwise manner following above-mentioned criteria. Out of 1345 sequence variations, 139 were monomorphic across 13 genotypes (Table 2). Concerning polymorphism status within each group, 699 sequence variations had same allele in 3 genotypes of susceptible group and at the same time, at least one resistant genotype had contrasting allele, whereas remaining 507 sequence variations were polymorphic across susceptible group of 3 genotypes. In the case of resistant group of 10 genotypes, 309 monomorphic and 897 polymorphic sequence variations were identified (Table 2). For further analysis, 699 sequence variations showing similar allele in susceptible genotypes and alternate allele in at least one resistant genotype were considered. From these 699 sequence variations, we zeroed-in on the 4 sequence variation showing alternate allele in all 10 resistant genotypes. Further, similar to FW, grading score was followed for remaining 695 sequence variations. Consequently, 6 more sequence variations were selected, of which top 4 sequence variations had alternate allele in 8 out of 10 resistant genotypes. Remaining 2 sequence variations were showing alternate allele in 7 genotypes out of 10 resistant genotypes (Supplementary Table 7). As a result, a set of 10 sequence variations was identified as the most informative markers. Interestingly, 4 of these 10 sequence variations were present in the candidate genomic regions of CcLG04 identified earlier (Saxena et al. 2017a; Singh et al. 2017). Two each from the remaining selected sequence variations were present in genomic region on CcLG02 and CcLG07, whereas one sequence variation each was present on CcLG06 and CcLG08. The functional annotation of selected 10 sequence variation included 4 intergenic and 6 genic variations. These genic variations were present in four genes including two genes, namely “*C*.*cajan*_20995” (2 intronic and 1 missense), “*C*.*cajan*_21801” (1 intronic) on CcLG04, *C*.*cajan_07858* (1 synonymous) on CcLG02 and *C*.*cajan_17341* (1 intronic) on CcLG07 (Supplementary Table 7).

### Development of Kompetitive allele-specific PCR (KASP) genotyping assay

Above-mentioned 20 sequence variations were selected to develop KASP genotyping assays. Following the filtering criteria as mentioned in the Methods section, genotyping assays were designed for all the 20 sequence variations. In order to validate these newly designed KASP assays, 74 pigeonpea genotypes including parents of segregating populations, released varieties and elite breeding material and 18 BC_1_F_1_ individual plants were used (Supplementary Table 4). As a result, 19 from the total 20 KASP assays designed were successfully amplified on the validation set of genotypes in two DNA concentrations. Based on the ability of a particular KASP assay in differentiating homozygote and heterozygote, all the KASP assays were characterized in four different categories (cat), i.e. **Cat1** (13 KASP assays: compact two homozygous clusters representing alleles in parents and one heterozygous cluster for hybrids are present), **Cat2** (5 KASP assays: able to form homozygous and heterozygous clusters but migrate very close together), **Cat3** (1 KASP assay: all three are scattered and close to each other; there were mismatches between two tested DNA concentrations; discrepancies in genotyping calls were due to dilution factor of DNA and presence of PCR inhibitors at high concentration) and **Cat4** (1 KASP assay: did not show amplification for any of the dilutions tested) (Supplementary Table 8).

### Polymorphism assessment and genetic relationships in pigeonpea genotypes

As described above, 74 genotypes were used for assessing the polymorphism of newly developed 19 KASP assays (Table 3). All the developed 19 KASP assays showed polymorphism across the 74 pigeonpea genotypes. The polymorphism information content (PIC) value of these markers ranged from 0.28 (snpCC00052, snpCC00067) to 0.37 (snpCC00055, snpCC00057, snpCC00058, snpCC00059, snpCC00060, snpCC00064) with an average of 0.35. While major allele frequency at the polymorphic loci ranged from 0.51 (snpCC00060) to 0.79 (snpCC00052), the expected heterozygosity (He) varied from 0.03 (snpCC00064) to 0.28 (snpCC00051) with an average of 0.1 per marker (Table 3).

**Table 3:** Allele frequency, heterozygosity and polymorphism information content of selected SNPs

Marker	CcLG	Position in genome (bp)	Major allele	Major allele frequency	Minor allele	Minor allele frequency	Heterozygosity	Polymorphism information content (PIC) value
snpCC00051	CcLG07	405411	A	0.71	–(Del)	0.29	0.28	0.32
snpCC00052	CcLG07	359129	G	0.79	–(Del)	0.21	0.08	0.28
snpCC00053	CcLG02	35442671	C	0.68	T	0.32	0.11	0.34
snpCC00054	CcLG02	35444865	T	0.68	C	0.32	0.11	0.34
snpCC00055	CcLG04	1311824	G	0.59	A	0.41	0.13	0.37
snpCC00056	CcLG04	1311853	G	0.59	A	0.41	0.14	0.36
snpCC00057	CcLG04	1311883	G	0.59	A	0.41	0.13	0.37
snpCC00058	CcLG08	6474381	C	0.57	T	0.43	0.11	0.37
snpCC00059	CcLG04	9064323	TTAA	0.54	–(Del)	0.46	0.09	0.37
snpCC00060	CcLG06	22373087	C	0.51	T	0.49	0.08	0.37
snpCC00061	CcLG11	40660145	G	0.63	T	0.37	0.08	0.36
snpCC00062	CcLG11	40660325	G	0.63	A	0.37	0.08	0.36
snpCC00063	CcLG11	40661720	G	0.62	A	0.38	0.08	0.36
snpCC00064	CcLG11	16084073	–(Del)	0.56	ATATGAA	0.44	0.03	0.37
snpCC00065	CcLG11	40660118	C	0.63	A	0.37	0.07	0.36
snpCC00067	CcLG04	9496888	–(Del)	0.78	CA	0.22	0.04	0.28
snpCC00068	CcLG10	4381948	–(Del)	0.71	A	0.29	0.12	0.32
snpCC00069	CcLG07	16759426	G	0.67	T	0.33	0.07	0.35
snpCC00070	CcLG08	10391962	A	0.74	G	0.26	0.07	0.32
Mean				0.64		0.36	0.1	0.35

**Fig. 2:**
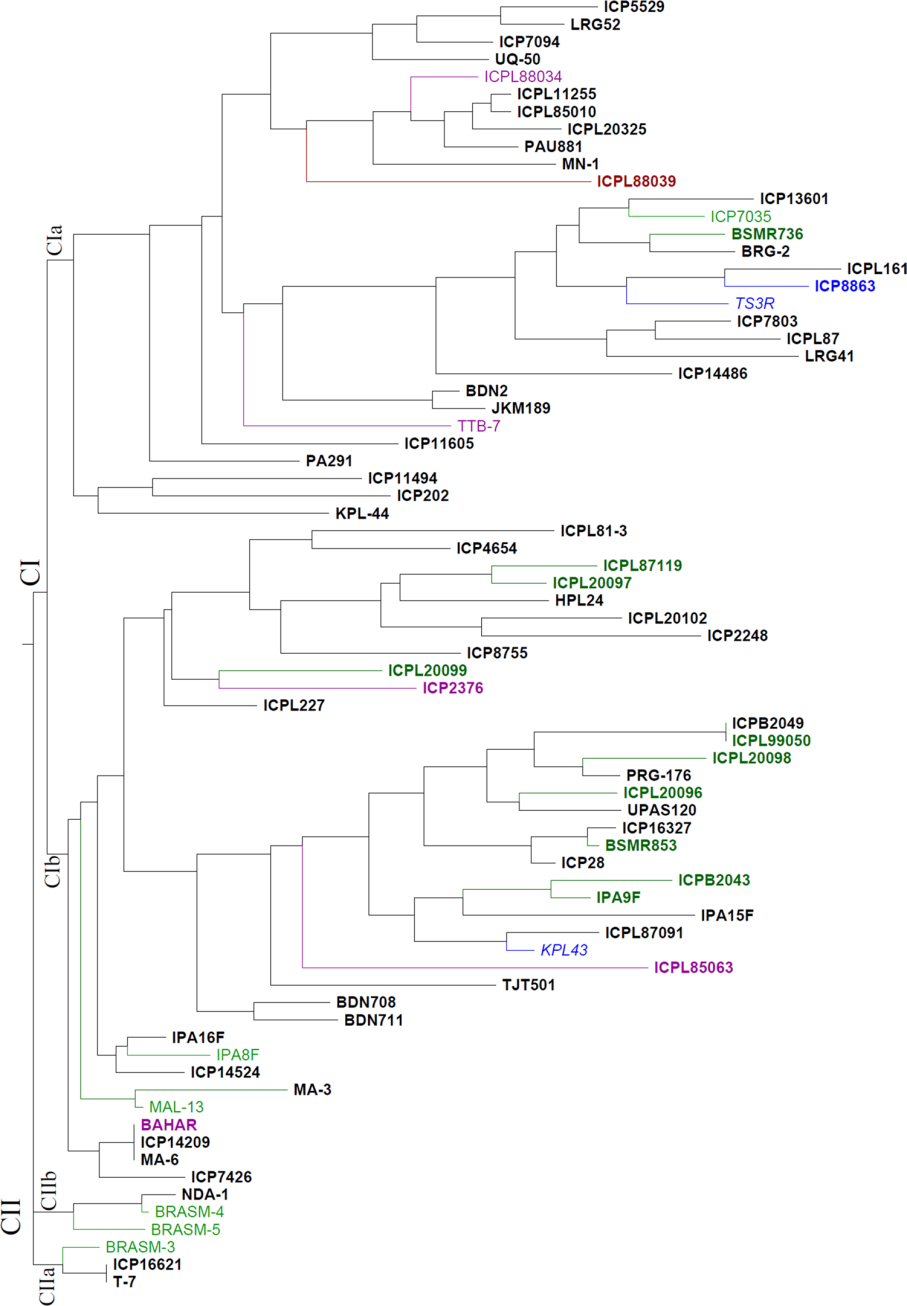
An overview on genetic relationships among 74 pigeonpea genotypes based on informative/diagnostic markers associated with resistance to Fusarium wilt (FW) and sterility mosaic diseases (SMD). The colour scheme for the names of genotypes has been followed as: genotypes with no phenotyping data available for FW and SMD (black); genotypes with phenotyping data available for both FW and SMD: (1) FW resistant and SMD resistant (dark green bold), (2) FW resistant and SMD susceptible (blue bold), (3) FW susceptible and SMD resistant (purple bold), (4) FW susceptible and SMD susceptible (red bold); genotypes with phenotyping data available for FW and not SMD: (1) FW resistant (blue italics), (2) FW susceptible (purple italics); genotypes with phenotyping data available for SMD and not FW: (1) SMD resistant (green normal). “C”: cluster (colour figure online)

Genotyping data obtained for all 19 polymorphic markers on 74 genotypes were used to prepare the genetic dissimilarity matrix for construction of dendrogram using DARWIN program. The dendrogram classified all genotypes into two main clusters (Fig. 2). Cluster I (CI) contained 68 genotypes, and cluster II (CII) had 6 genotypes. Under each of the main clusters, genotypes were grouped further into sub-clusters. For instance, CI was divided into two sub-clusters, namely CIa with 30 genotypes and CIb with 38 genotypes, whereas CII had two sub-clusters, namely CIIa and CIIb with 3 genotypes in each (Fig. 2). Majority of the resistant genotypes for FW and SMD were grouped in CI, and only 3 SMD resistant genotypes were grouped in CII (BRASM-3 in CIIa and BRASM-4 and BRASM-5 in CIIb). In CIa, 4 resistant genotypes (BSMR736 resistant to both FW and SMD, ICP 7035 resistant to SMD and ICP 8863 and TS3R resistant to FW) were grouped with other susceptible genotypes. CIb had most of the resistant genotypes including 8 genotypes resistant to both SMD and FW, 5 genotypes resistant to SMD and 1 genotype resistant to FW. Further, genotyping data were analyzed to estimate the efficiency of diagnostic markers in detecting resistant and susceptible alleles across 74 genotypes (Supplementary Table 4). Therefore, genotyping data obtained by total 19 polymorphic markers were divided into two sub-datasets, i.e. genotyping data on nine markers for estimating their efficiency in detecting resistant and susceptible alleles for FW and genotyping data on ten markers for estimating their efficiency in detecting resistant and susceptible alleles for SMD. Out of 74 genotypes, phenotyping data were available on 18 genotypes for FW and 20 genotypes for SMD (Supplementary Table 4). In the case of FW, phenotyping data classified 18 genotypes as five susceptible, one moderate resistant and 12 resistant genotypes. While correlating nine markers data with phenotyping data on FW, in all the susceptible genotypes no resistant allele was present. In the moderate FW resistant genotype (“ICPL 85063”), three resistant, two susceptible and four heterozygous alleles were found. In FW resistant genotypes (12), a range of 3–9 resistant alleles were present. Three FW resistant genotypes (ICPL 20096, ICPL 20098 and ICPL 99050) have shown the presence of all nine resistant alleles (Supplementary Table 4), whereas SMD phenotyping data classified 20 genotypes into two susceptible and 18 resistant genotypes. While correlating ten markers data with SMD phenotyping data, both susceptible genotypes (ICP 8863 and ICPL 88039) had no resistant alleles. In SMD resistant genotypes (18), a range of 5–10 resistant alleles were present. Five SMD resistant genotypes (BSMR 736, ICPL 20098, IPA 9F, MAL-13 and PRG-176) have shown the presence of all 10 resistant alleles (Supplementary Table 4). In brief, above-mentioned results have shown the usefulness of selected markers in detecting FW/ SMD susceptible and resistant alleles in tested genotypes. As a next step, all the 19 markers were also tested for their application in marker assisted back-crossing (MABC). A total of 18 BC_1_F_1_s and their crossing parents (BDN 711 × ICPL 20096) were genotyped with all 19 markers. BDN 711 is moderately resistant to FW and resistant to SMD. In high disease pressure of FW, BDN 711 has shown susceptibility to FW. Therefore, ICPL 20096 has been used as donor parent in MABC to enhance the FW resistance in BDN 711. The genotyping data on BDN 711 have shown the presence of six susceptible and three resistant alleles for FW, whereas ICPL 20096 has all nine resistant alleles for FW. Though both the parents had nine resistant and one susceptible alleles for SMD, but one marker in each genotype had different alleles. For instance, “snpCC00058” had SMD susceptible allele in BDN 711 but resistant allele in ICPL 20096. Likewise, “snpCC00059” had SMD susceptible allele in ICPL 20096 but resistant allele in BDN 711 (Supplementary Table 4). Subsequently, polymorphic markers on parents and their relationships with FW/SMD resistance were used to select most desirable BC_1_F_1_s with better combinations of alleles.

#### Geographical distribution of resistant alleles

**Fig. 3:**
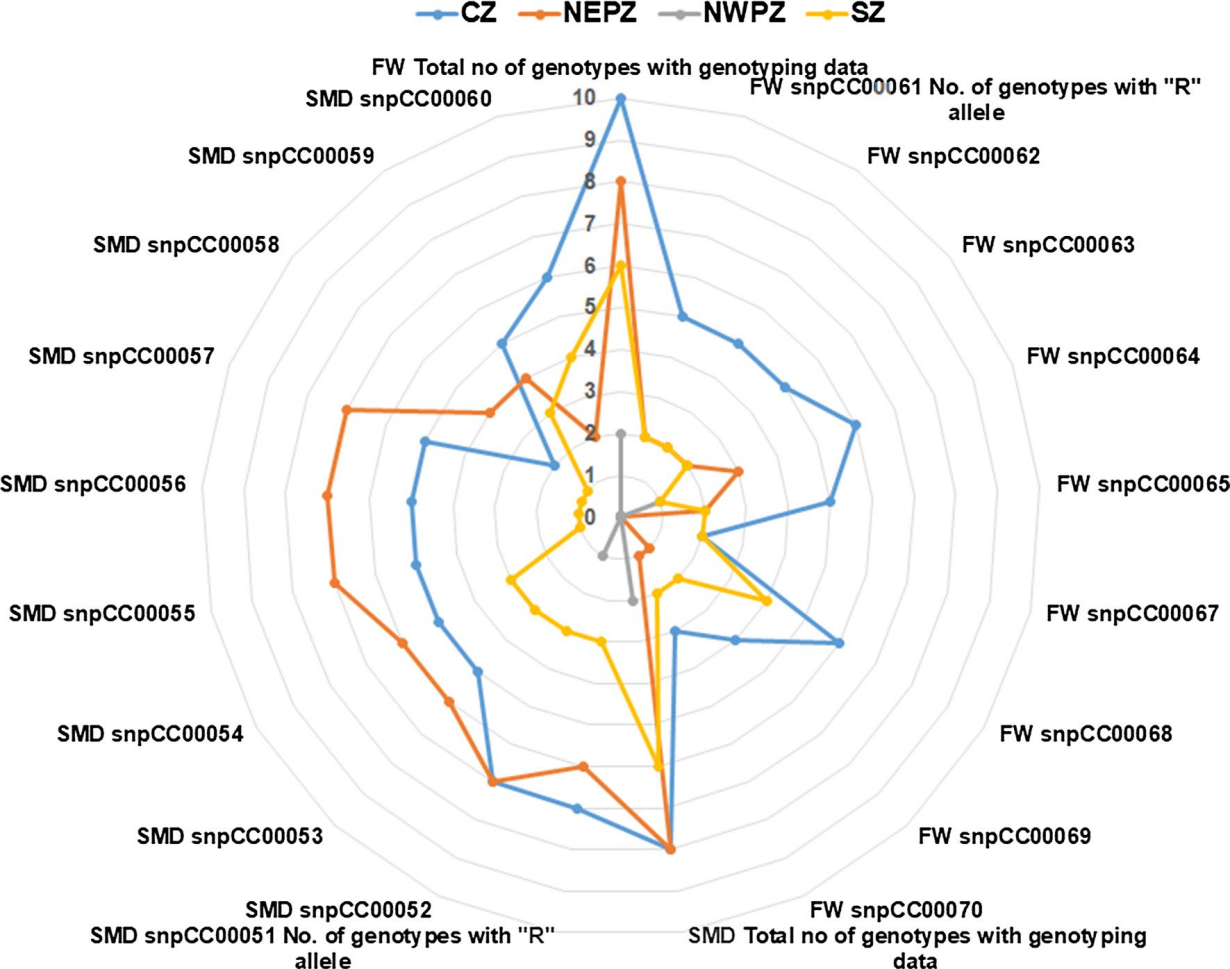
Geography-based distribution of resistant alleles in pigeonpea genotypes for Fusarium wilt (FW) and sterility mosaic disease (SMD). Adaptation/cultivation zones in India taken under consideration based on available information for the genotypes used in the present study are Central Zone (CZ, blue line), South Zone (SZ, yellow line), North-east Plain Zone (NEPZ, orange line) and North-west Plain Zone (NWPZ, grey line). Right side represents the presence of FW-associated resistant alleles in genotypes from CZ, SZ, NEPZ and NWPZ. Left side represents the presence of SMD-associated resistant alleles in genotypes from CZ, SZ, NEPZ and NWPZ

In order to assess geographical distribution of resistant alleles identified in the present study, a subset of 26 genotypes was selected on the basis of information available on zone of adaptation/cultivation (Supplementary Table 9). Two genotypes were considered in two zones of adaptation, i.e. ICPL 87119 in both central zone (CZ) and south zone (SZ) and ICPL 88039 in both northeast plain zone (NEPZ) and northwest plain zone (NWPZ). The distribution frequencies of resistant alleles coming from 19 diagnostic markers varied in different zones of adaptation. For 9 resistant alleles to FW, genotypes have shown the presence of 1–7 resistant alleles in CZ, 0–5 resistant alleles in NEPZ, 0–1 in NWPZ resistant alleles and 0–7 resistant alleles in SZ. Similarly, for 10 resistant alleles to SMD, genotypes have shown the presence of 0–10 resistant alleles in CZ and NEPZ, 0–1 in NWPZ resistant alleles and 0–7 resistant alleles in SZ. All 19 resistant alleles were also checked for their presence in different zones (Fig. 3). In the case of FW, CZ has the highest number of resistant alleles, followed by SZ and NEPZ. For SMD, NEPZ has the highest number of resistant alleles, followed by CZ and SZ. Above-mentioned results based on limited sets of genotypes have clearly shown the existence of alleles variations in resistant genotypes for FW and SMD diseases in different adaptation zones. However, to get more clear picture on variations for the FW and SMD resistant alleles in genotypes, it is recommended to use all the cultivars grown in adaptation zones.

## Discussion

In recent times, most of the genomics studies have been conducted using next-generation sequencing (NGS)-based genotyping methodologies or high-density genotyping platforms (Singh et al. 2016; Roorkiwal et al. 2013; Saxena et al. 2012; Kassa et al. 2012). Similarly, in the case of pigeonpea, a number of datasets have been produced using NGS (Varshney et al. 2012) and *Cajanus* SNP array (Saxena et al. 2018; Singh et al. 2020). These datasets have been used in several studies, for instance GBS for trait mapping (Saxena et al. 2017a, b, c, 2020), WGS for trait mapping, gene discovery, diversity, evolutionary analysis (Varshney et al. 2017; Singh et al. 2016), SNP array data for diversity analysis, trait mapping (Yadav et al. 2019), etc. Most of these studies, if not all, remained stand alone or as a next step of previously conducted one or two studies. However, most of these above-mentioned studies were based on defined nucleotide (SNPs and Indels) positions in the genome and provided an opportunity to build up new strategy by combining their outputs, datasets, etc., in a thoughtful manner to achieve major goals. Following this hypothesis, we have used the previous findings in FW and SMD mapping (Saxena et al. 2017a, b, c; Singh et al. 2016, 2017), high-density genotyping data on elite breeding genotypes (Saxena et al. 2018; Kumar et al. 2016; Varshney et al. 2017) and trait phenotyping data to develop most informative/robust sets of markers in the present study.

In the present approach, different data sets have been combined to identify 20 most informative sequence variations for diagnosis of FW and SMD resistant alleles. Designated panel of genotypes with known reaction to FW and SMD diseases has confirmed the presence of susceptible and resistant alleles (Supplementary Table 7). However, successful KASP assays could be designed for 19 markers. These 19 markers (9 for FW and 10 for SMD) have been proposed to use as a diagnostic kit to enable crop improvement teams to reduce the effects of FW and SMD on pigeonpea yield. In order to use this kit for diagnostic purpose, if KASP genotyping facilities are not available locally, just leaf samples can be send to testing laboratories. The cost for per sample genotype with 10 markers including DNA extraction is ~ 2.5 US$ with a minimum of 384 total samples. However, genotyping cost may vary depending on the total number of samples. Depending on the diseases variability, we would like to propose to the crop improvement teams to apply two steps selection in tested material with this newly developed kit. The ***first step*** in selection is to apply negative selection and discard samples/genotypes showing presence of susceptible alleles. This is due to possibilities of existence of other resistant loci in remaining samples/genotypes which might have not covered in the present kit. The ***second step*** of selection would select those lines which have passed from the negative selection and carrying maximum number of favourable alleles covered in the present kit. The second step of selection could be applied to enhance the frequencies of favourable alleles in the elite breeding material.

This approach has also provided major genomic segments involved in diseases resistance. For instance, in the case of FW genomic region at CcLG11 (gene *C.cajan*_03691) showed major role in resistance. The gene “*C.cajan*_03691” was predicted to encode “Pumilio homolog 6, (APUM-6)”. The PUF RNA-binding proteins (referred also as PUMILIO proteins) are known to be conserved family in all eukaryotes. However, these PUF proteins show variability in their repeat number, position and amino acid sequence (Zhang and Muench 2015) and represent cases of gene duplication events (Tam et al. 2010). Functional data have suggested that several APUMs play important roles in biotic and abiotic stress responses (Francischini and Quaggio 2009; Huh et al. 2012). Another key gene identified on CcLG07 (*C*.*cajan_18888*) codes for “Allene oxide cyclase” protein. A previous study on the mutant lines for the gene encoding allene oxide cyclase (OsAOC) in rice has shown its role in the defence response to the blast fungus *Magnaporthe oryzae* (Riemann et al. 2013). Similarly in SMD, we found a major role of CcLG04 (gene *C*.*cajan_20995*) in imparting resistance (Supplementary Table 7). The gene “*C*.*cajan*_20995” has been shown to be translating “Myb protein 1 (DdTom1)”. Myb proteins are known to function as transcription factors with their ability to bind DNA. In plants species, MYB families play an important role in controlling plant development, metabolism (Dubos et al. 2010) and response to biotic stresses (Gao et al. 2016). The functions of *MYB* genes have been explored in a number of plants species, such as rice (*Oryza sativa*) (Yanhui et al. 2006), petunia (*Petunia hybrida*), snapdragon (*Antirrhinum majus*), grapevine (*Vitis vinifera* L.), poplar (*Populus tremuloides*) and apple (*Malus domestica*) (Dubos et al. 2010). Additionally, three genes *C*.*cajan_07858* (CcLG02), *C*.*cajan_21801* (CcLG04) and *C*.*cajan_17341* (CcLG07) code for “ABC transporter G family”, “Myb-related protein” and “probable receptor-like protein kinase”, respectively, are known for their role in biotic stress resistance in plants. These findings will be crucial not only for understanding the diversity and functionality of the candidate genes but also for determining the mechanism of resistance to FW and SMD.

## Conclusions

Comprehensive data analysis has successfully identified 9 robust markers for FW resistance and 10 robust markers for SMD resistance in pigeonpea. These markers have been converted into a diagnostic kit for their routine use in crop improvement programs focusing on the development of FW and SMD resistant genotypes. Further, we identified two genes for FW and four genes for SMD resistance that offers new opportunities for assign functional role and understanding their participation in molecular mechanisms underlying diseases resistance in pigeonpea. The allele diversity of the robust markers across the resistant and susceptible genotypes will be critical in obtaining novel allelic combinations imparting greater resistance against diseases. We expect that in future further functional validation, including uncovering epistatic interactions of these alleles, would fast-track the development of FW and SMD resistant pigeonpea varieties.

## Electronic supplementary material

Below is the link to the electronic supplementary material.

Supplementary file1 (XLSX 9 kb)

Supplementary file2 (XLSX 18 kb)

Supplementary file3 (XLSX 16 kb)

Supplementary file4 (XLSX 21 kb)

Supplementary file5 (XLSX 10 kb)

Supplementary file6 (XLSX 362 kb)

Supplementary file7 (XLSX 12 kb)

Supplementary file8 (XLSX 15 kb)

Supplementary file9 (XLSX 13 kb)
